# Clinical Benefit With PARP Inhibitor for Pathogenic Germline *FANCA*-Mutated Relapsed Epithelial Ovarian Cancer: A Case Report

**DOI:** 10.3389/fonc.2022.778545

**Published:** 2022-02-25

**Authors:** Bing Qian, Wenshu Leng, Zhengqing Yan, Jin Lu, Shiqing Chen, Huan Yi, Zhi Jiang

**Affiliations:** ^1^ Jiangsu Cancer Hospital, Jiangsu Institute of Cancer Research, The Affiliated Cancer Hospital of Nanjing Medical University, Nanjing, China; ^2^ Department of Medical Affairs, The Medical Department, 3D Medicines Inc., Shanghai, China

**Keywords:** PARP inhibitor, ovarian cancer, germline, FANCA, clinical benefit

## Abstract

**Background:**

PARP inhibitors have been approved as targeted therapy for BRCA-deficient metastatic ovarian cancer (OC). Fanconi anemia complementation group A (*FANCA*), one of the homologous recombination repair pathway genes, is a susceptibility gene to breast cancer and OC. Therefore, it is interesting to investigate whether germline *FANCA*-mutated relapsed epithelial OC could achieve clinical benefit from the treatment of PARP inhibitor.

**Case Presentation:**

A 49-year-old female patient without a family history of cancer was diagnosed with epithelial OC. This patient underwent surgical resection plus platinum-based treatment twice in 2016 and 2018, successively. After the second relapse in July 2019, the patient underwent another radical resection. The next-generation sequencing analysis results revealed a germline *FANCA* mutation in the tumor tissue. Subsequently, the third-line treatment of liposomal doxorubicin hydrochloride plus lobaplatin was administrated for five cycles with the patient’s consent. Then, oral niraparib (200 mg daily) was given for maintenance treatment. During the follow-up, no evidence of tumor recurrence was observed. Currently, the survival with no evidence of disease has already exceeded 21 months, and the treatment is still going on.

**Conclusions:**

This case highlighted that OC patients harboring pathogenic gene alterations in the homologous recombination pathway might achieve clinical benefit from PARP inhibitors, which should be confirmed in further studies.

## Introduction

Ovarian cancer (OC) is the most common cause of death from gynecological cancer among women worldwide (1). Approximately 80% of patients with newly diagnosed OC have a response to platinum-based chemotherapy. However, most patients would relapse and achieve limited benefits from subsequent therapies. The median progression-free survival (PFS) after the first, second, third, fourth, and fifth relapse was 10.2 [95% confidence interval (CI) 9.6–10.7], 6.4 (5.9–7.0), 5.6 (4.8–6.2), 4.4 (3.7–4.9), and 4.1 (3.0–5.1) months, respectively (2).

PARP inhibitors, such as olaparib and niraparib, are new treatment strategies for BRCA 1/2 altered OC and other cancers (3). BRCA1/2-deficient cells utilize error-prone DNA repair pathways, causing increased genomic instability, which might be responsible for their sensitivity to DNA-damaging agents. Niraparib is an oral, highly selective PARP1 and PARP2 inhibitor (4). In the NOVA trial, all patients who received niraparib had a significantly longer PFS than those who received placebo. Notably, BRCA-mutated patients could achieve more benefits from PARP inhibitors than non-BRCA-mutated patients (5). Similar to BRCA1/2, some other “BRCAness” genes (e.g., ATM, PALB2, and FANC) also play key roles in homologous recombination pathway (6, 7). According to previous reports, a subset of patients harboring deleterious gene mutations in the non-BRCA homologous recombination repair pathway (HRRm) might benefit from PARP inhibitor (8). Furthermore, for patients with non-BRCA HRRm, the extent of benefit from PARP inhibitors was different (9). Thus, identifying non-BRCA HRRm is critical for precision treatment and survival management for OC patients.

Fanconi anemia complementation group A (*FANCA*) is associated with Fanconi anaemia, a rare autosomal recessive disorder characterized by congenital abnormalities, bone marrow failure, and predisposition to malignancy. Recently, *FANCA* has emerged mainly as a susceptibility gene to breast cancer and OC (10, 11). The single-strand annealing activity of *FANCA* plays a direct role in double-strand break (DSB) repair (12, 13). Preclinical studies demonstrated an association between *FANCA* mutated cells and sensitivity to PARP inhibitors (7). Compared with control isogenic wild-type cells, *FANCA*-deficient mouse fibroblast cells demonstrated greater sensitivity to PARP inhibitors. Another study reported that *FANCA* p.S1088F could induce sensitivity to olaparib *in vitro* in cancer cell lines or *in vivo* in patient-derived xenografts (14). Besides this, PARP inhibitors demonstrate promising results in *FANCA*-altered metastatic castration-resistant prostate cancer (mCRPC) (6, 15, 16). Herein we reported the first case of a germline *FANCA*-mutated relapsed epithelial OC who achieved clinical benefit from PARP inhibitors.

## Background

**Figure 1:**
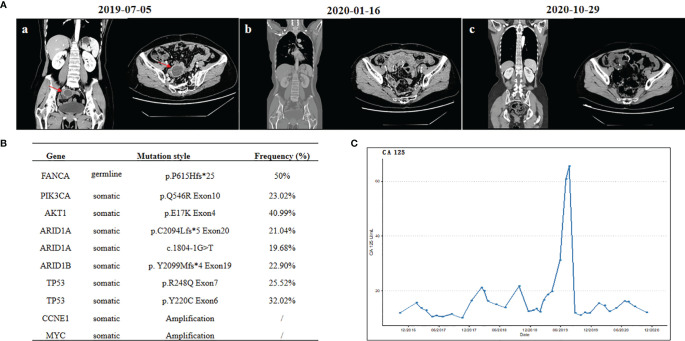
**(A)** Computed tomography scans: (a) before cytoreductive surgery, (b) before niraparib treatment, and (c) after 9 months of niraparib treatment. **(B)** List of deleterious or likely deleterious somatic variations by next-generation sequencing in ovarian carcinoma. **(C)** Changes of serum tumor marker levels of carbohydrate antigen 125.

A 49-year-old female patient was admitted to the hospital due to a growing left adnexal mass in August 2016. She had no family history of cancer. The results of laboratory tests showed normal levels of carbohydrate antigen (CA) 125, alpha-fetoprotein, carcinoembryonic antigen (CEA), and human epididymis protein 4. However, the CA 19-9 level was up to 62.65 U/ml. The ultrasound results revealed a 10.9 × 8.5-cm cystic mass on the left ovary. Thus, she underwent a series of surgical procedures, including total hysterectomy, bilateral salpingo-oophorectomy, and omentectomy (R0 resection). Intraperitoneal perfusion of carboplatin was given during the operation. The postoperative pathology confirmed a moderately differentiated ovarian endometrioid adenocarcinoma. After the operation, the patient underwent four cycles of paclitaxel/carboplatin. Given that the CA19-9 level did not drop to the normal range after chemotherapy, carboplatin was replaced by lobaplatin since the fifth cycle. The patient underwent one cycle of paclitaxel/lobaplatin. This patient achieved complete response with normal CA19-9 level. After the first relapse with a pelvic metastasis in October 2018, debulking surgery was performed (R0 resection). Postoperative pathology revealed high-grade serous OC. The CA125 level was 21.7 U/ml after operation (normal range, 0–35 U/ml). The patient was administrated with a second-line chemotherapy of paclitaxel/lobaplatin from November 2018 to February 2019. However, CT scanning revealed a cystic-appearing solid mass in the pelvic cavity (**Figure 1A**), and the CA125 level was increased up to 60+ U/ml in July 2019, suggesting that she developed tumor progression after the paclitaxel/platinum-free interval of 4 months in the second-line therapy. Subsequently, she underwent radical surgery with R0 resection. A 7-cm-sized mass was seen in the rectovaginal pouch during the operation, and a postoperative pathological examination confirmed a poorly differentiated ovarian carcinoma.

To further explore the genomic features, both the surgical specimen and the matched white blood cell (WBC) sample underwent next-generation sequencing analysis based on a pan-cancer 733-gene panel (3D Medicines, China). Germline mutation was identified from WBC sequencing results. The mutation profiling of this patient is summarized in **Figure 1B**. The results suggest that this patient harbored germline *FANCA* p. P615Hfs*25 heterozygous mutation and somatic *PIK3CA*, *AKT1*, *ARID1A*, *ARID1B*, and *TP53* mutations, with a high homologous recombination deficiency (HRD) score of 35 (cutoff = 30). It was noted that the HRD score is defined as the unweighted numeric sum of the loss of heterozygosity score, telomeric allelic imbalance score, and large-scale state transition score, according to 3DMed-HRD algorithm as previously described (17). The HRD score threshold of 30 is predefined by analyzing the HRD scores in a Chinese training cohort of breast and ovarian cancer patients with known BRCA1/2 status and identifying a cutoff with 95% sensitivity to detect those tumors with BRCA1/2mutation. Previous works suggested that *FANCA* could increase the sensitivity to DNA-damaging agents, such as platinum (14, 18). Since August 2019, the third-line treatment of liposomal doxorubicin hydrochloride plus lobaplatin (five cycles) was administrated with patient consent. No evidence of disease (NED) was observed during the third-line chemotherapy. Subsequently, oral niraparib (200 mg daily) was administered for maintenance treatment. During the follow-up, no evidence of tumor recurrence was observed (**Figure 1A**), and the CA125 level remained within the normal range (**Figure 1C**). No adverse events were observed during niraparib treatment. Currently, the survival with NED has already exceeded 21 months, and the period of niraparib treatment has been more than 15 months. The close follow-up is still going on.

## Discussion

In this report, we presented a case of relapsed epithelial OC harboring germline *FANCA* p. P615Hfs*25 heterozygous mutation who responded well to the niraparib treatment with PFS of over 21 months. In this case, different histologic subtypes are confirmed after operation, which reflect the tumor heterogeneity. Pathological transformation was also observed in a previous OC case, whose pathology transformed to undifferentiated small cell carcinoma from adenocarcinoma (19). In this case, the CA19-9 level was kept at a high level at diagnosis, while the CA125 level was increased to a high level during the second recurrence. Although the CA19-9 level was usually elevated in gastrointestinal cancer, it could also be detected in the blood of some OC patients (20). This case supported that the combination detection of serum CA125, CA19-9, and CEA might have higher sensitivity and specificity compared to a single serum marker. The paclitaxel/platinum-free interval is less than 6 months during the second-line treatment, suggesting that paclitaxel or platinum resistance was developed during the second recurrence. Notably, current clinical evidence could neither support nor deny the benefit of extending platinum-free interval in patients with recurrent ovarian cancer (21). Thus, lobaplatin-based strategy is still administrated as the third-line treatment in this case.

Besides BRCA1/2, pathogenic gene variants involving Fanconi anemia have been reported as candidate ovarian cancer-predisposing genes (10, 22). Though no family cancer history is observed in this case, the relationship between pathogenic germline gene variants and OC should be further studied in the future. About 0.23% of patients harbor germline or somatic *FANCA* mutations in the TCGA OC cohort (23). Its single-strand annealing activity plays a direct role in DSB repair (12, 13). To the best of our knowledge, several clinical trials demonstrated that *FANCA*-mutated patients respond to PARP inhibitors *via* a synthetic lethality mechanism. In phase 2 TRITON2 study, one mCRPC patient with *FANCA* mutation had complete radiographic and prostate-specific antigen (PSA) responses to the PARP inhibitor rucaparib (6). In phase 2 GALAHAD study evaluating niraparib in mCRPC, tumor radiographic response was observed in two patients with alterations in *FANCA* (15). The phase 2 TOPARP-B study presented another mCRPC patient harboring a *FANCA* mutation who had PSA responses after olaparib monotherapy (16). How *FANCA* mutations affect the genomic instability and the efficiency of PARP inhibitors in *FANCA*-mutated OC patients should be investigated in the future. Though no secondary hits were found on *FANCA* gene, this patient had a high HRD score of 35 (cutoff = 30), which might be able to explain the significant clinical benefit from niraparib.

Based on their great clinical benefits to OC patients, PARP inhibitors such as olaparib, rucaparib, and niraparib had been approved for OC maintenance treatment after platinum-based chemotherapy. A previous study demonstrated that the median PFS of third-line chemotherapy in relapsed patients was only 5.6 (4.8–6.2) months (2). When PARP inhibitors are used for maintenance therapy in relapsed OC patients, the median PFS of germline BRCA-mutated patients is up to 21 months, according to the NOVA trial results. In contrast, the PFS is just 9.3 months in these relapsed OC patients with wild-type BRCA (5). In this case, the patient harboring pathogenic *FANCA* achieved clinical benefit with PFS of over 21 months, almost equivalent to that of BRCA-mutated patients with PARP inhibitors. Consistent with a previous study (24), such result highlighted that PAPRP inhibitor was efficacious not only in BRCA-mutated patients but also in patients with unknown alterations. Given the nature of case reports, larger cohort studies should be investigated to further confirm such conclusions.

## Concluding Remarks

In conclusion, we presented the first case of one relapsed epithelial OC harboring a germline *FANCA* mutation who achieved an impressive PFS after niraparib treatment. This case highlighted that OC patients carrying pathogenic HRRm might achieve the best outcome from PARP inhibitors. Such a conclusion should be confirmed in further studies.

## Ethics Statement

Written informed consent was obtained from the patient before clinical samples were collected. Consent to publication was also obtained from the patient. The patient was informed of the test results.

## Author Contributions

ZJ contributed to planning and organization. BQ collected clinical data and supervised the findings of this work. JL and WL aided in the data collection and supervision. ZY, SC, and HY analyzed the results and prepared the manuscript. All authors contributed to the article and approved the submitted version

## Conflict of Interest

ZY, SC, and YH were employed by the company 3D Medicines Inc.

The remaining authors declare that the research was conducted in the absence of any commercial or financial relationships that could be construed as a potential conflict of interest.

## Publisher’s Note

All claims expressed in this article are solely those of the authors and do not necessarily represent those of their affiliated organizations, or those of the publisher, the editors and the reviewers. Any product that may be evaluated in this article, or claim that may be made by its manufacturer, is not guaranteed or endorsed by the publisher.
